# High degree of mitochondrial gene heterogeneity in the bat tick species *Ixodes vespertilionis*, *I. ariadnae* and *I. simplex* from Eurasia

**DOI:** 10.1186/s13071-015-1056-2

**Published:** 2015-09-17

**Authors:** Sándor Hornok, Agustín Estrada-Peña, Jenő Kontschán, Olivier Plantard, Bernd Kunz, Andrei D. Mihalca, Adora Thabah, Snežana Tomanović, Jelena Burazerović, Nóra Takács, Tamás Görföl, Péter Estók, Vuong Tan Tu, Krisztina Szőke, Isabel G. Fernández de Mera, José de la Fuente, Mamoru Takahashi, Takeo Yamauchi, Ai Takano

**Affiliations:** Department of Parasitology and Zoology, Faculty of Veterinary Science, Szent István University, Budapest, Hungary; Department of Animal Pathology, University of Zaragoza, Zaragoza, Spain; Plant Protection Institute, Centre for Agricultural Research, Hungarian Academy of Sciences, Budapest, Hungary; INRA, Biologie, Epidémiologie et Analyse de Risque en santé animale, Nantes, France; LUNAM Université, Oniris, Ecole nationale vétérinaire, agroalimentaire et de l’alimentation Nantes-Atlantique, Nantes, France; Independent researcher, Langenburg, Germany; Department of Parasitology and Parasitic Diseases, University of Agricultural Sciences and Veterinary Medicine, Cluj-Napoca, Romania; Solar View Cottage, Upper Mawprem, Shillong, Meghalaya, India; Laboratory for Medical Entomology, Centre of Exellence for Food and Vector-Borne Zoonoses, Institute for Medical Research, University of Belgrade, Belgrade, Serbia; Chair of Animal Ecology and Zoogeography, Institute of Zoology, Faculty of Biology, University of Belgrade, Belgrade, Serbia; Department of Zoology, Hungarian Natural History Museum, Budapest, Hungary; Institute for Veterinary Medical Research, Centre for Agricultural Research, Hungarian Academy of Sciences, Budapest, Hungary; Department of Zoology, Eszterházy Károly College, Eger, Hungary; Institute of Ecology and Biological Resources, Vietnam Academy of Science and Technology, Hanoi, Vietnam; SaBio. Instituto de Investigación en Recursos Cinegéticos IREC, Ciudad Real, Spain; Center for Veterinary Health Sciences, Oklahoma State University, Stillwater, USA; Department of Anesthesiology, Saitama Medical University, Iruma-gun, Japan; Toyama Institute of Health, Imizu, Toyama Japan; Department of Veterinary Medicine, Joint Faculty of Veterinary Medicine, Yamaguchi University, Yamaguchi, Japan

**Keywords:** Chiroptera, *Ixodes*, Cytochrome oxidase gene, 12S rRNA, 16S rRNA

## Abstract

**Background:**

Phylogeographical studies allow precise genetic comparison of specimens, which were collected over large geographical ranges and belong to the same or closely related animal species. These methods have also been used to compare ticks of veterinary-medical importance. However, relevant data are missing in the case of ixodid ticks of bats, despite (1) the vast geographical range of both *Ixodes vespertilionis* and *Ixodes simplex*, and (2) the considerable uncertainty in their taxonomy, which is currently unresolvable by morphological clues.

**Methods:**

In the present study 21 ticks were selected from collections or were freshly removed from bats or cave walls in six European and four Asian countries. The DNA was extracted and PCRs were performed to amplify part of the cytochrome oxidase I (COI), 16S and 12S rDNA genes, followed by sequencing for identification and molecular-phylogenetic comparison.

**Results:**

No morphological differences were observed between *Ixodes vespertilionis* specimens from Spain and from other parts of Europe, but corresponding genotypes had only 94.6 % COI sequence identity. An *I. vespertilionis* specimen collected in Vietnam was different both morphologically and genetically (i.e. with only 84.1 % COI sequence identity in comparison with *I. vespertilionis* from Europe). Two ticks (collected in Vietnam and in Japan) formed a monophyletic clade and shared morphological features with *I. ariadnae*, recently described and hitherto only reported in Europe. In addition, two Asiatic specimens of *I. simplex* were shown to differ markedly from European genotypes of the same species. Phylogenetic relationships of ticks showed similar clustering patterns with those of their associated bat host species.

**Conclusions:**

Although all three ixodid bat tick species evaluated in the present study appear to be widespread in Eurasia, they exhibit pronounced genetic differences. Data of this study also reflect that *I. vespertilionis* may represent a species complex.

## Background

Bats (Chiroptera) are the second largest order of mammals. Besides the high number of their species, they form the largest known mammalian aggregations (colonies) in which they may reach extremely high individual numbers [1]. Thus, frequently being the most abundant group of the local vertebrate fauna, their epidemiological role as pathogen reservoirs is also increasingly recognized [2]. Owing to their adaptability, i.e. large variety of their foraging habitats (forests, plains, along water surfaces, urban areas), roosting places (caves, tree holes, buildings) and prey animals (insects, arachnids, even vertebrates), microbats occur on all continents, except the Antarctica [1].

Apart from birds, bats are the only vertebrates that are able to actively fly. Consequently, during their (seasonal) migration, bats may even cover a few hundred kilometers. However, (unlike birds) they are unable to cross high mountain ranges, frequently rendering their populations geographically isolated. Such isolation may serve as the basis of bat speciation [3, 4] and may also have an impact on the evolution of bat parasites, including ticks.

Hard ticks (Acari: Ixodidae) that are specific to bats are only known to occur in the Old World. In Eurasia, for more than a century, only three species of ixodid ticks were known to infest bats: *Ixodes vespertilionis* Koch, *I. simplex* Neumann and *I. kopsteini* Oudemans [5, 6]. However, recently a new species, *I. ariadnae* Hornok has been described and molecularly characterized [7]. These four species appear to differ in their host preferences, i.e. *I. vespertilionis* occurs predominantly on *Rhinolophus* spp. [5, 8], *I. ariadnae* mainly on *Myotis* spp. [7], whereas *I. simplex* on *Miniopterus schreibersii* [5] and *I. kopsteini* on Molossidae [6]. Thus, the geographical range limit, the migration habit, the various habitat preferences and hibernating behavior of relevant bat species will significantly influence the geographical distribution of their ticks. Both *I. vespertilionis* and *I. simplex* are among the tick species with the largest known geographical range, encompassing much of the Old World (from Europe to the south in Africa and Australia, and to the east in Asia, including Japan). However, despite this, phylogeographical studies have not yet been conducted to investigate the morphological and/or genetic uniformity of these two tick species throughout their vast range.

Formerly, allopatric genotypes of *I. vespertilionis* were shown to exist between distant caves within a country [7]. To extend the scope of such observations, the present study was initiated in order to evaluate the mitochondrial gene heterogeneity of ixodid bat ticks over a larger range in the Old World, including ticks that showed the morphological characteristics of *I. vespertilionis*, *I. simplex* and *I. ariadnae*. In Southern and Eastern Asian countries involved in the present study the sample number was limited, because ixodid bat ticks are rare (e.g. [9]).

The 5’ region of the mitochondrial cytochrome oxidase subunit I (COI) gene was chosen as the primary target for molecular analyses. This is the standard marker for tick species identification by DNA barcoding, with confirmatory or supportive value of 16S and 12S rDNA sequences [10]. These methods were found to be suitable to distinguish between the three ixodid bat tick species that occur in Europe [11]. Therefore, phylogenetic analyses in the present study are based on the COI gene, with additional testing of part of the 16S and/or 12S genes in the case of highly divergent specimens.

## Methods

Ticks were collected from bats captured for ringing and monitoring purposes, or from cave walls near bats (Table 1). All specimens were stored in 70 % ethanol. The morphology of ticks was compared according to the length/shape of palps, shape and index (length/width) of the scutum, density of alloscutal setae, arrangement of coxal setae. DNA was extracted with the QIAamp DNA Mini Kit (Qiagen, Hilden, Germany) according to the manufacturer’s instructions; when possible, only from 1–4 legs.

**Table 1:** Collection data of bat ticks processed in this study. DNA extraction was attempted from all samples, but GenBank accession number is provided only in the case of successful COI gene amplification. 16S and 12S rDNA sequence accession numbers are provided in the text

tick species acc. to morphology	tick stage/sex	host species	date of collection	place of collection: country (city or region)	COI GenBank accession number
*I. vespertilionis*	F	*Myotis mystacinus*	20-05-2010	Serbia (Zlot)	KR902765
	N	*Rhinolophus hipposideros*	03-05-2013	Serbia (Dimitrovgrad)	KR902764
	F, F	(cave wall)	1907^a^	Serbia (Novi Pazar)	-
	N	*Rhinolophus ferrumequinum*	07-09-2014	Bosnia-Herzegovina (Šipovo)	KR902763
	N	*Rhinolophus* sp.	1899^a^	Bosnia-Herzegovina (43.2° N, 17.8°E)	-
	N	*Rhinolophus euryale*	22-09-2013	Montenegro (Rijeka Crnojevića)	KR902766
	F	*Rhinolophus hipposideros*	19-09-1913^a^	Czech Republic (Hranice na Morave)	-
	M, F	near *Rhinolophus hipposideros*	21-02-2008	France (Ruillé en Champagne)	KR902757-8
	F	*Rhinolophus* sp.	ca. 1992	Spain - Vasque Country	KR902759
	N	*Rhinolophus* sp.	ca. 1992	Spain - Vasque Country	KR902760
	N	*Rhinolophus* sp.	ca. 1992	Spain - Vasque Country	KR902761
	L	*Rhinolophus* sp.	ca. 1992	Spain - Vasque Country	KR902762
	M	(cave wall)	1890^a^	Russia (Caucasus - Labinsk)	-
	N	*Rhinolophus affinis*	20-10-2014	Vietnam (Cao Bang - Phia Oac)	KR902756
*I. ariadnae*-like	L	*Myotis* n. sp. (undescribed)	17-10-2014	Vietnam (Thanh Hoa - Ban Vin)	KR902767
	F	*Murina leucogaster*	09-03-2013	Japan (Okayama)	LC036330
*I. simplex*	F	near *Miniopterus schreibersii*	09-07-2008	France (Rancogne)	KR902768
	N	*Miniopterus magnater*	19-02-2015	India (Meghalaya - Jaintia Hills)	KR902769
	L	*Miniopterus fuliginosus*	09-09-2013	Japan (Wakayama)	LC055099

^a^kindly provided by the Natural History Museum of Berlin. Abbreviations: M-male, F-female, N-nymph, L-larva

In the case of Japanese samples, the COI PCR, that amplifies an up to 710 bp long fragment of the gene with the primers HCO2198 (5’-TAA ACT TCA GGG TGA CCA AAA AAT CA-3’) and LCO1490 (5’-GGT CAA CAA ATC ATA AAG ATA TTG G-3’), was performed according to [12]. Two further PCRs were used to amplify an approx. 460 bp fragment of the 16S rDNA gene of Ixodidae with the primers 16S + 1 (5’-CTG CTC AAT GAT TTT TTA AAT TGC TGT GG-3’) and 16S-1 (5’-CCG GTC TGA ACT CAG ATC AAG T-3’) [13]; and to amplify an approx. 420 bp fragment of the 12S rDNA gene of hard ticks with the primers 12S + 1 (5’-TAC TAT GTT ACG ACT TA-3’) and 12S-1 (5’-AAA CTA GGA TTA GAT ACC C-3’) [14].

For the remaining samples PCR conditions were slightly modified. The COI primers were used in a reaction volume of 25 μl, containing 1 U (0.2 μl) HotStarTaq Plus DNA polymerase, 2.5 μl 10× CoralLoad Reaction buffer (including 15 mM MgCl_2_), 0.5 μl PCR nucleotid Mix (0.2 mM each), 0.5 μl (1 μM final concentration) of each primer, 15.8 μl ddH_2_O and 5 μl template DNA. For amplification, an initial denaturation step at 95 °C for 5 min was followed by 40 cycles of denaturation at 94 °C for 40 s, annealing at 48 °C for 1 min and extension at 72 °C for 1 min. Final extension was performed at 72 °C for 10 min. In the case of selected specimens that were highly divergent according to the COI analysis, the 16S and 12S rDNA gene PCRs were also performed. Reaction components other than primers, as well as cycling conditions were the same, except for annealing at 51 °C.

PCR products were electrophoresed (for visualization), purified and sequenced. The sequences were submitted to GenBank (accession numbers KR902756-77). Phylogenetic analyses were conducted as reported [11], including that for bat host species of ticks based on cytochrome *b* sequences retrieved from GenBank. Phylogenetic trees were compared in the R development framework [15].

The partial 12S rDNA gene amplification and sequencing with the above primer pair was not successful in the case of the *I. vespertilionis* nymph from Vietnam and *I. simplex* from India (Table 1), consistently with the selective success of primers reported by Norris *et al.* [14].

### Ethical approval

Authorization for bat capture was provided by the National Inspectorate for Environment, Nature and Water. Bat banding licence numbers are TMF-493/3/2005 (TG) and 59/2003 (PE).

## Results

### Comparison of ixodid bat ticks from Europe

**Fig. 1:**
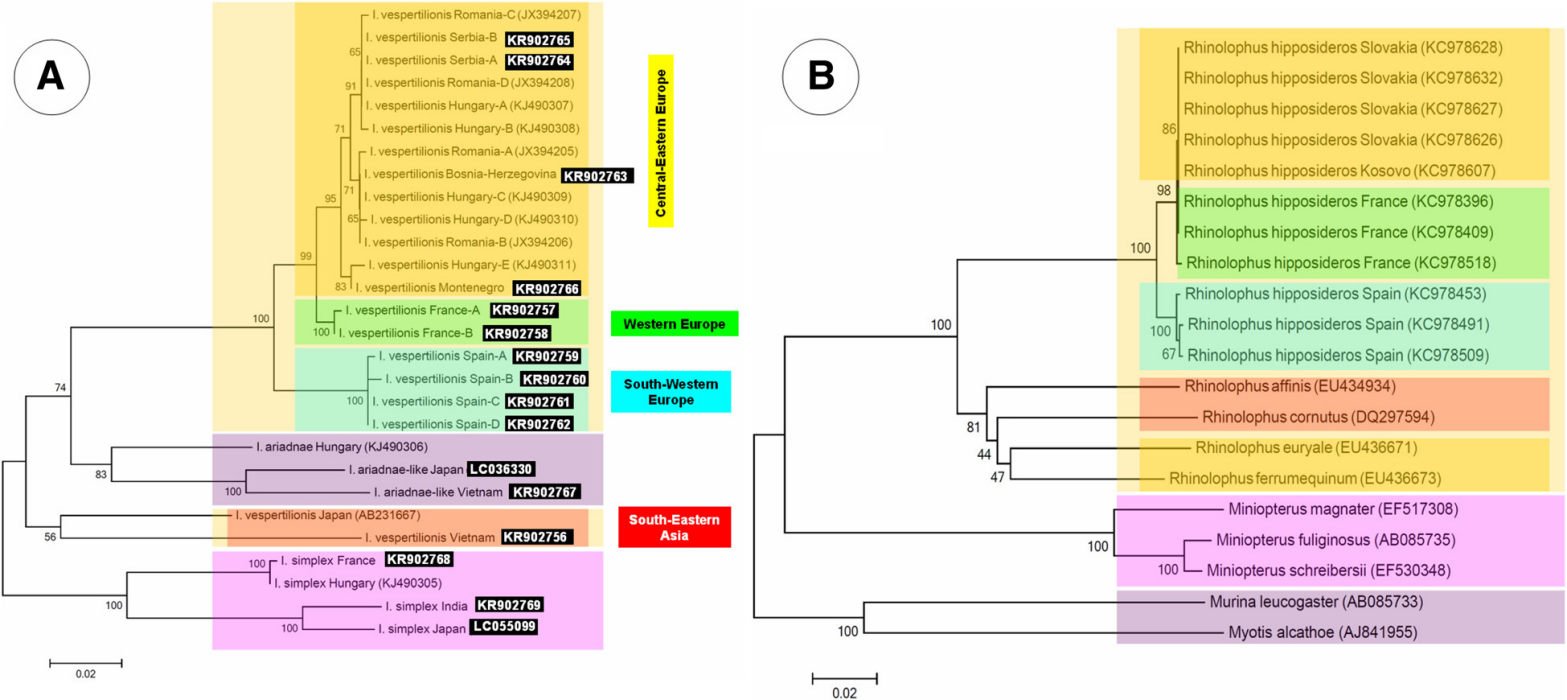
**a** Phylogenetic relationships of bat ticks collected in the present study (accession number in inverse colour) based on COI gene and related data from the GenBank. Light yellow background indicates isolates of *I. vespertilionis*, with overlaying background colours according to geographical regions as indicated. Purple background colour stands for genotypes of *I. ariadnae*, and pink for those of *I. simplex*. **b** Phylogenetic relationships of bat species that were hosts of the tick species (according to the colour code of the tick phylogenetic tree) analysed in the present study. Branch lengths correlate to the number of substitutions inferred according to the scale shown

No morphological differences were noted between specimens of *I. vespertilionis* collected in four different countries of Central-Eastern (CE) Europe. The amplified part of the COI gene showed a maximum of eight nucleotide difference, i.e. 98.7 % identity between isolates. In the phylogenetic tree these genotypes clustered together, but separately from Western European *I. vespertilionis* specimens collected in France (Fig. 1a). The latter differed with up to 15 nucleotides, and in that case had only 97.6 % sequence identity with CE European genotypes.

The specimens of *I. vespertilionis* from Spain also appeared to be morphologically identical to those from CE Europe. However, the COI genotypes of these South-Western European isolates showed up to 34 nucleotide differences when compared to CE Europe, i.e. only 94.6 % identity between genotypes of the two regions. In the phylogenetic analysis the South-Western European isolates formed a distinct cluster, a sister group of all other evaluated European specimens (Fig. 1a), similarly to genotypes of *R. hipposideros*, the main host of *I. vespertilionis* (Fig. 1b).

**Fig. 2:**
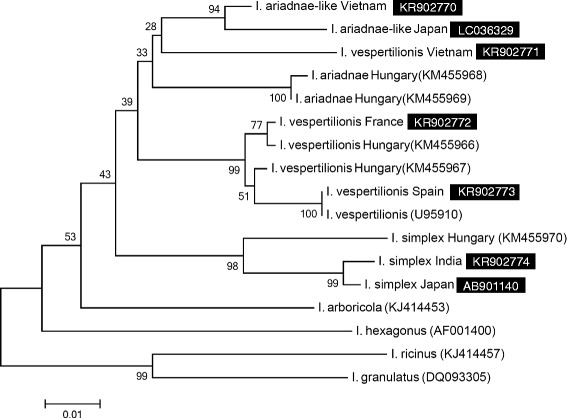
Phylogenetic comparison of 16S rDNA sequences of bat tick genotypes identified in the present study (inverse colour) and other sequences from the GenBank. Branch lengths correlate to the number of substitutions inferred according to the scale shown

Accordingly, based on the amplified part of its 16S rDNA gene, the French isolate (accession number: KR902772) clustered together with CE European isolates, but separately from the Spanish one (KR902773), although the bootstrap support for the latter was low (Fig. 2). The distinct phylogenetic position of a Spanish *I. vespertilionis* isolate was further confirmed by analysis of part of the 12S rDNA gene, because the genotype from France (KR902776) showed three nucleotide differences from (i.e. 99 % sequence identity to) specimens collected in CE Europe, whereas the Spanish isolate (KR902777) had nine nucleotide differences and thus only 97.5 % sequence identity.

### Comparison of ixodid bat ticks from Southern and Eastern Asia

**Fig. 3:**
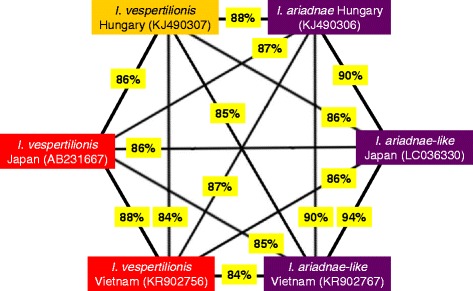
The approximated degree of COI sequence identity between highly divergent genotypes of *I. vespertilionis* and *I. ariadnae*. Background colour of species names corresponds to the one used on Fig. 1a. Three sequences are from other studies (KJ490306-7: [7]; AB231667: [19])

The *I. vespertilionis* nymph collected in Vietnam had convex posterolateral margin of the scutum, as contrasted to the concave shape in the case of European specimens. The COI sequence of this tick (KR902756) also had the highest level of intraspecific genetic divergence observed in this study: it differed from the CE European *I. vespertilionis* genotypes with up to 101 nucleotides, i.e. showed only 84.1 % sequence identity and clustered distantly on the phylogenetic tree (Fig. 1a). This nymph from Vietnam had the closest sequence identity (88 %) to a formerly published Japanese genotype (AB231667) (Fig. 3). Based on its partial 16S rDNA gene sequence (KR902771) this tick clustered separately from all European *I. vespertilionis* specimens and as the sister group of the two Asiatic ticks resembling *I. ariadnae* (Fig. 2).

Another tick was collected from an undescribed species of *Myotis* in Vietnam. This larva showed morphological similarities to *I. ariadnae*. Consistently with this, the COI sequence of this specimen (KR902767) had the highest (89.5 %) identity with the CE European *I. ariadnae* genotype (62 nucleotide differences), and only 85.2 % identity with the CE European *I. vespertilionis* genotypes (i.e. up to 87 nucleotide differences). Based on COI phylogenetic analysis this *I. ariadnae*-like genotype from Vietnam clustered close to *I. ariadnae*, but separately from *I. vespertilionis* collected in CE Europe (Fig. 1a). This finding was only partly confirmed by the analysis of the 16S gene (although poorly supported by low bootstrap values), because the Vietnamese *I. ariadnae*-like specimen (KR902770) clustered separately from CE European specimens of both *I. ariadnae* and *I. vespertilionis* (Fig. 2). The partial 12S gene sequence of this larva (KR902775) showed only 12–14 nucleotide differences from (96–96.6 % identity with) *I. ariadnae*, whereas 19–20 nucleotide differences from (94.3–94.6 % identity with) *I. vespertilionis*.

One female tick collected from *Murina leucogaster* in Japan also resembled morphologically to *I. ariadnae*. The COI sequence of this specimen (LC036330) differed from the CE European *I. ariadnae* genotype in 65 nucleotides (amounting to 89.7 % identity), but from CE European *I. vespertilionis* genotypes in up to 85 nucleotides (meaning only 86.4 % identity). The COI phylogenetic analysis showed that this *I. ariadnae*-like genotype from Japan clustered together with the above *I. ariadnae*-like tick from Vietnam and close to *I. ariadnae* from Hungary, but separately from *I. vespertilionis* collected in CE Europe (Fig. 1a). This finding was also confirmed by the phylogenetic tree based on the amplified part of the 16S gene (LC036329, Fig. 2). The partial 12S gene sequence of this tick (LC036328) showed 15–17 nucleotide differences from (95.4–95.9 % identity with) *I. ariadnae*, whereas 20–21 nucleotide differences from (94.3–94.6 % identity with) *I. vespertilionis*. The genetic distances based on partial COI sequences between highly divergent *I. vespertilionis* and *I. ariadnae* genotypes are summarized in Fig. 3.

One nymph, collected from *Miniopterus magnater* in India, showed a similar morphology to *I. simplex*. However, the partial COI sequence of this specimen (KR902769) had 55–58 nucleotide differences from two European genotypes (i.e. 90.8–91 % identity), from which it also clustered apart in the phylogenetic analysis (Fig. 1a). Similarly, the separation of this Indian *I. simplex* genotype (KR902774) from the Hungarian one was supported by high bootstrap value in the 16S phylogenetic analysis (Fig. 2).

A female *I. simplex*, collected from *Mi. fuliginosus* in Japan, was also included in the present study. The partial COI sequence of this isolate (LC055099) had only 25 nucleotide differences from (i.e. 96 % identity with) the above genotype from India, and in the phylogenetic analysis these two Asiatic *I. simplex* specimens clustered together, but separately from two European (French and Hungarian) genotypes (Fig. 1a). The same grouping pattern was seen on the basis of the partial 16S rDNA gene (AB901140) of this isolate (Fig. 2). At the same time, its partial 12S rDNA sequence (LC055100) showed 24 nucleotide differences from (93.6 % identity with) *I. simplex* from Hungary.

## Discussion

The results of the present study can be interpreted in the light of evolutionary factors and events as well as ecological traits that influence the intra- and inter-specific genetic diversity of animal populations, in this case of ticks and their bat hosts. In this context the geographical range of bat species, geographical barriers and glacial periods (that may isolate related bat populations) may be particularly important as driving forces of disruptive selection. We acknowledge that the reduced number of samples and stages used in the present study prevent conclusions about the precise taxonomic status of collected specimens. However, the low number of samples is compensated by an adequate representation of the territories and a high similarity of the samples within-sites. We could not explicitly calculate the amount of phylogenetic diversity at each node of the phylogenetic tree because the presence of singletons, or isolated taxa that produced only a single edge in the tree. However, the relative distances among the tips of the tree are an indirect measure of the similarity within and between sites.

The main host of *I. vespertilionis* in Europe is *R. hipposideros* [5, 8]. During post-glacial periods this bat species recolonized central and northern Europe from refugia either in the Balkan or the northern Mediterranean (Southern France) [16]. This may account for the relative genetic homogeneity of CE European and French *I. vespertilionis* isolates, as reported here.

Considering all *I. vespertilionis* analyzed in the present study from Europe, specimens collected in Spain were shown to differ markedly (and to cluster apart phylogenetically) from other evaluated genotypes. Probably the most important underlying factor of this phenomenon is that bat hosts of *I. vespertilionis* represent isolated populations on the Iberian peninsula, prevented from mixing with northern populations by the Pyrenees which act as a barrier to gene flow [16]. This is also well reflected by the phylogenetic position of *R. hipposideros* from Spain and CE Europe (Fig. 1b). The level of sequence divergence between Spanish and CE European genotypes of *I. vespertilionis* (5.4 % in the COI gene) approached the proposed sequence difference as species boundary for ticks (6.1 % COI: [10]). In contrast to this, another member of the genus, *I. ricinus* was demonstrated to show only 0.5 % COI sequence divergence between specimens from CE Europe or France and Spain (e.g. GU074908 or GU074941 vs. GU074910: [17]), explained by gene flows due to dispersal and continuous exchange of ticks between populations connected by migrating hosts.

The homologies of COI sequences were reported to be above 93.9 % within a species, and below 94.4 % between species of ixodid genera [18]. Similarly, in another study the percentage of species boundary was deemed to be 6.1 % sequence divergence for the COI gene, only in exceptional cases amounting to higher values [10]. Accordingly, in comparison with *I. vespertilionis* genotypes from Europe (KR902757-66), the high (16 %) genetic divergence of *I. vespertilionis* from Vietnam (KR902756) and of a similar genotype in Japan (AB231667), together with their well separated and distant phylogenetic position, suggest that they probably represent a distinct tick species. This is partly confirmed by the morphological difference observed between the European and the Vietnamese variant. In addition, the degree of partial COI sequence divergence between the European and Vietnamese *I. vespertilionis* genotypes (101 nucleotide differences, i.e. 84.1 % identity) was similar to that observed between the latter and *I. ricinus* (e.g. in comparison with KF197132: 102 nucleotide differences, i.e. 83.9 % identity). At the same time, the two South-East Asiatic genotypes (KR902756, AB231667) clustered together and showed 88 % similarity to each other (Fig. 3).

The host of the *I. vespertilionis* nymph collected in the present study in Vietnam was *R. affinis*, and of that recorded previously in Japan was *R. cornutus* ([19]: AB231667). Based on phylogenetic analysis of mitochondrial (cytochrome *b*) sequences these two bat species clustered separately from other representatives of the genus (that harbored ticks in the present study), similarly to the phylogenetic position of associated ticks (Fig. 1a-b). These two Asiatic *Rhinolophus* spp. are closely related phylogenetically to *R. ferrumequinum* (Fig. 1b). which appears to be the most important host of *I. vespertilionis* in Japan and Eastern Asia [5, 9]. Although the Palearctic geographical range of *R. ferrumequinum* extends from Western Europe to Eastern Asia, the spatial separation of glacial refugia and temporal shift of recolonization events (in Europe vs. East Asia) may have helped to maintain the genetic diversity in both the bat hosts (as reported by Flanders *et al.* [20]) and their *I. vespertilionis* ticks (as supported by data of the present study).

Findings of this study also attest, for the first time, the existence of genetic and morphologic variants of bat ticks in Asia that are most closely related to *I. ariadnae*, hitherto only recognized in Europe. *Myotis* spp. are among the preferred hosts of *I. ariadnae* ([7]; S. Hornok, unpublished observations), and one *I. ariadnae*-like genotype of the present study was collected from a *Myotis* sp. in Vietnam. Murininae, including *Murina leucogaster* from which the *I. ariadnae*-like genotype was collected in Japan, are also closely related to *Myotis* spp. [4]. This lineage (consisting of hosts of *I. ariadnae* and similar genotypes) shows clearly a separate position on the bat phylogenetic tree (Fig. 1b).

The taxonomy of the preferred host of *I. simplex*, *Miniopterus schreibersii*, has recently undergone revision. While previously it was considered as a uniform species widely distributed across the Old World, it turned out to be a complex comprising several species [21]. Among them, in the present study *I. simplex* was collected from both *Mi. magnater* (in India) and *Mi. fuliginosus* (in Japan), allowing phylogeographical comparison with the ticks collected from *Mi. schreibersii* in Europe. In the phylogenetic analysis not only did the four *I. simplex* genotypes cluster separately from other bat tick isolates (Fig. 1a), but also their three respective bat host species from other bat species involved in the present study (Fig. 1b). Again, glaciation and resultant geographic isolation might be considered as major mechanisms underlying the genetic differentiation of these closely related Asiatic *Miniopterus* spp. [21], and may have also served as the basis for the relative genetic heterogeneity (96 % COI sequence similarity) of their ticks as demonstrated in the present study.

It has been suggested that in the case of an exophilic, generalist tick species, like *I. persulcatus* (which has a similarly broad, but northern Eurasian geographical range), glaciation events may have caused the loss of significant genetic variation because of genetic mixing during refuge formation [22]. On the contrary, populations of bat ticks and their hosts in the southern parts of Asia may have remained relatively unaffected by ice age(s), maintaining drivers in formerly initiated species divergence. Moreover, separation from other parts of Eurasia during the glacial periods might fulfill the role of geographical barrier formation for South-Eastern Asia, where the biodiversity could be maintained or was even enhanced during ice ages [23].

## Conclusions

Strict morphological criteria of all tick developmental stages and genotyping from a larger number of samples may help to clarify the precise taxonomical status of highly divergent genetic variants of ixodid bat ticks reported here. This will also compensate for the lack of voucher specimens in the case of some South East Asiatic bat tick genotypes tested molecularly in the present study or reported previously. Data of this study also reflect that *I. vespertilionis* may represent a species complex, given the strong bootstrap support for the exclusion of two specimens from Japan and Vietnam from the clade that contains all specimens from Europe.
